# A hybrid trust computing approach for IoT using social similarity and machine learning

**DOI:** 10.1371/journal.pone.0265658

**Published:** 2022-07-28

**Authors:** Amr M. T. Ali-Eldin

**Affiliations:** Computer Engineering and Control Systems Department, Faculty of Engineering, Mansoura University, Mansoura, Egypt; University of Pisa, ITALY

## Abstract

Every year, millions of new devices are added to the Internet of things, which has both great benefits and serious security risks for user data privacy. It is the device owners’ responsibility to ensure that the ownership settings of Internet of things devices are maintained, allowing them to communicate with other user devices autonomously. The ultimate goal of the future Internet of Things is for it to be able to make decisions on its own, without the need for human intervention. Therefore, trust computing and prediction have become more vital in the processing and handling of data as well as in the delivery of services. In this paper, we compute trust in social IoT scenarios using a hybrid approach that combines a distributed computation technique and a global machine learning approach. The approach considers social similarity while assessing other users’ ratings and utilize a cloud-based architecture. Further, we propose a dynamic way to aggregate the different computed trust values. According to the results of the experimental work, it is shown that the proposed approaches outperform related work. Besides, it is shown that the use of machine learning provides slightly better performance than the computing model. Both proposed approaches were found successful in degrading malicious ratings without the need for more complex algorithms.

## Introduction

The Internet of Things (IoT) is gaining popularity due to its low energy consumption and increased economic value as a result of the proliferation of smart devices and recent advancements in telecommunications [1]. The Internet of Things is a network of sensors, tools, and devices that are connected via a network and capable of sending and receiving messages without human intervention [2, 3]. Security concerns are growing as the Internet of Things (IoT) is rapidly being deployed [4]. Security issues about wireless sensor networks (WSNs), machine to machine systems (M2Ms), cyber-physical systems (CPSs), and computer networks are still being raised in the context of IoT [1].

Additional considerations for security solutions for IoT devices are the limitations imposed by their restrictive architectural designs. IoT in healthcare and smart cities are examples of IoT paradigms where security threats and vulnerabilities are still being explored, and there is presently no ideal solution available [5, 6]. IoT environments allow for the communication of millions of different entities or artefacts, making standard security components unable to deal with cyber-attacks on a widespread and large scale [7]. Further, IoT environments may contain misbehaving users or be vulnerable to malicious attacks. Thus, the goal of this research is to determine the trustworthiness of an IoT object acting as a service provider (SP) in the IoT cyberspace by linking it to the trustworthiness of another IoT object acting as a service requestor (SR) [8]. Previous interactions with other IoT devices, as well as the service provider’s social relationships with the service requestors, are all being considered in this study.

The social implications of computing and IoT, such as how to provide trustworthy services, are key topics to consider when developing technology [9]. Concerns have been raised regarding IoT potential negative implications. This is because of the large amounts of data collected from the physical layers that must be sufficiently reliable. Obtaining the wide trust qualities required for IoT data processing is a complex challenge that does not have an easy solution at this time to the best of our knowledge. Internet of Things (IoT) services, on the other hand, demand a significant amount of data processing and mining. This fact increases the likelihood of someone’s privacy being violated. Offering contextually aware and personalized services through networked devices while also protecting user privacy is a difficult and complex problem [9–11].

The goal of the future Internet of Things is to make autonomous decisions without the need for human participation. Consequently, trust has become increasingly important in the processing and handling of data and services [12]. Current trust models place a high value on how much a client trusts and appreciates other people in their organization. In the end, however, we conclude that establishing a trust model that can address all of the difficulties that arise in social IoT systems is a difficult mission [13].

In the dictionary, trust can be defined as confidence in the dignity, strength, capability, or assuredness of a person or thing. According to [14], defining trust is quite complex as it appears in many disciplines and thus can have different meanings. Trust is assessed either by the user himself based on his or her perception of others or by the help of peers’ experiences in the case of unknown entities [15]. One of the most serious issues, with trust computing approaches, is users’ lack of familiarity with traditional trust models. Another issue to consider is the widespread use of out-of-date ratings, as well as the consequences of making incorrect assessment decisions or even being held accountable for fraudulent conduct. Although there are several alternatives to trust computing, they are extremely difficult to build and require a significant amount of computing resources to function properly [11]. Given the scarcity of available resources and the immaturity of IoT devices [8], more tailored computational models are required to provide reliable assessments [16].

One method of assisting in the prevention of Internet of Things (IoT) threats is to employ machine learning (ML) algorithms. However, because of the difficulty and power consumption associated with machine learning, not many studies have been conducted on its application to IoT trust management and security [17]. One of the main topics in machine learning, classification refers to the case when we train a model to use the features to predict an appropriate label that estimates the probability of the observed occurrence belonging to each of several classes. Supervised machine learning involves teaching an algorithm to identify and predict certain features [18]. Supervised learning methods include support vector machines (SVM), regression trees, linear networks, convolutional neural networks, deep belief networks, and recurrent neural networks. Unsupervised learning is used to classify unlabelled data into user-defined clusters by identifying common patterns. The most frequent unsupervised learning technique is clustering, which is like classification except that the training data has no predetermined class labels. An example of unsupervised network management algorithms is K-means clustering.

In this paper, we propose a hybrid approach for trust computing in IoT based on a computation model using social similarity and machine learning approaches. The proposed approach is considered hybrid as it combines two different approaches: a distributed approach for trust computing and a cloud-based machine learning one. Given the limited computing capabilities of IoT devices to process more complicated queries and machine learning classification algorithms used by this research, we propose a cloud-based architecture to execute the proposed machine learning algorithms to provide some global features that can be reused by users. In [19], a cloud-based model was proposed based on calculating social similarity. In this work, we include in trust computation other factors like IoT device proximity, quality of service, and user interactions. We further propose a dynamic trust aggregation approach. Furthermore, we provide high-level details of the proposed architecture. The main contribution of this paper is (1) the computation of trust in IoT combining machine learning and computational techniques with dynamic trust aggregation. (2) using social similarity among users and highest trusted users’ rates in degrading malicious and untrusted users’ ratings.

The paper is structured as follows: The next section discusses related work. Section III presents the proposed trust computational mechanisms. Section IV presents the proposed trust computing architecture together with its components. Experimental work is discussed together with obtained results in section V. Proposed models performance is evaluated in section VI while the paper is concluded in Section VII.

## Related work

With the development of the concept of incorporating social aspects into IoT, the emergence of a rapidly rising trend known as the ’Social Internet of Things’ was heralded (SIoT). By forming social relationships for the benefit of their owners, wireless IoT devices in smart cities can intelligently enhance information exchange and service discovery by connecting to the internet [20].

There are various sorts of trust computation models that have been examined in the literature for trust computation [12]. Most trust measures in the literature predicted that some variables, such as self-experience and the level of acceptability of service providers, would help individuals evaluate their level of confidence [21–24]. Some studies [25, 26], utilize aggregate values supplied by others within a population. Other ways for assessing trust have been proposed, such as negotiating trust using an authentication-based approach to access control [27].

There are two types of IoT trust management protocols: distributed solutions and centralized solutions. The work of [28] is an example of distributed trust management solutions, while examples of centralized solutions include [28–30]. The most challenging topic in distributed trust protocols is how to scale with enormous numbers of devices, such as those seen in IoT systems [29]. A key question is whether centralized trust-based resource management strategies will be successful in the face of the proliferation of complex Internet of things devices. A faulty forecast of trust will result from inconsistent interpretations of the social and interactional ties between Internet of Things devices. Because the cloud is unable to physically monitor these social links, it needs to collect relationship data from IoT devices on a dynamic basis to function properly. In addition, the large number of Internet of things devices connected to the cloud will waste a significant amount of energy because it will have an impact on the Internet’s communication function [29].

To undertake case study assessments of IoT cybersecurity policy and to use at the federal level in the United States, Chatfield et al. [31] developed a framework for IoT-enabled smart government performance, which was used by the United States federal government. Cicirelli et al. [32] proposed an Internet of Things (IoT)-based smart ecosystem modelling approach. The approach is built on a three-layered architecture that includes some abstractions that are well-suited for the development of IoT-based ecosystems and can utilize computational resources located at the network’s edge or in the cloud. A context-aware dispatcher framework for Internet of Things controlled systems is proposed by Amin et al. [2]. They put their method into practice during the fault management process in electric power distribution systems. Khan et al. [33] presented a context-aware, low-power, intelligent SmartHome architecture (CLPiSmartHome). In their proposal, they suggest a communication paradigm and architecture that would allow all electronic devices to communicate with one another using a centralized platform service. A conceptual model developed by Yildirim and Ali-Eldin [34] specifies elements that influence consumers’ intentions to adopt wearable Internet of Things devices at their place of employment.

Many recent pieces of research discussed and analysed security issues associated with IoT environments [1, 4, 35–41]. There has been substantial research into trust management in a range of domains, including economics, sociology, and computer science. When it comes to computer science, trust management is primarily concerned with issues of security and privacy. The trustor quantifies the qualitative or quantitative property of a trustee, and the trustor quantifies the belief as applying to a certain job, environment, and period, as defined by Jayasinghe et al. [12]. In a trust relationship, the trustor is the object that initiates an interaction with the trustee, which is another object that supplies the trustor with a service or specific knowledge. When it comes to trust management on IoT, available material is scarce. PeerTrust [28] is an online credibility engine for peer-to-peer networks that allows users to verify each other’s identities. The underlying concept is to present the buyer with the highest-quality recommendations, regardless of whether they are trustworthy. When determining whether a recommender is trustworthy, it considers additional factors that influence trustworthiness such as the transaction context, community context, and the recommender’s credibility. This allows it to discriminate between trustworthy and untrustworthy input.

A further feature of trust in the S-IoT is adaptive trustworthiness, in which trust rating is established by a combination of subjective and objective judgments. Rafey et al. [42] propose a social trust model that is bound by the environment in which it is used. When it comes to the study of trust, their paradigm places a strong emphasis on trust and trustworthiness. Nitti et al. [43] presented a server-based mechanism for regulating trust in the S-IoT, which was implemented in a prototype. Because it relied on a single system, it is vulnerable to cyber-attacks such as distributed denial of service (DDoS) and man-in-the-middle attacks [28] which are designed to disrupt the system. Increasingly, Sybil attacks represent additional potential attacks that not only boost spam and advertisements but also serve as distribution points for malware and phishing sites that can be used for cybercrime [28]. Complexity and vulnerability to performance concerns may represent the main challenges for the distributed scenario [29]. Therefore, a more effective mechanism for establishing the trustworthiness of an IoT device in cloud-based IoT networks is needed.

Machine learning (ML) algorithms are one method of assisting in the prevention of Internet of Things (IoT) attacks [33]. However, because of the difficulty and power consumption associated with machine learning, few studies have been conducted on its application to IoT trust management. In the literature, such as in [39], Bayes’ theorem is employed as a measure of trustworthiness due to its extensive functionality, which includes features like rapid updating and flexibility. In this study, we seek to quantify trust features and integrate them into a final judgment using a clustering strategy that groups extracted trust features and then the use of a KNN classifier to classify new trust requests.

## Proposed trust computing mechanism

IoT systems can create lots of data for a variety of applications. These devices interact in the same way as human relationships do, by utilizing established social features. It is possible to disseminate the values of social characteristics across IoT and consumer endpoints. As soon as the interaction is completed, the trust value of each user has changed accordingly. Then trust can be pooled across many people. It is necessary to aggregate all other users’ evaluations of a given user to calculate the trust value associated with it. Each user maintains a local list of trust values for other users with whom it has come into contact. To calculate trust in this context, one must combine two computed values: self-trust (also known as a direct trust or subjective trust), and indirect trust (also known as global or objective reputation), both of which are computed values. Self-trust or direct trust refers to how the user perceives others’ trustworthiness based on his or her previous interactions with them. In the event of unfamiliar service providers or users, indirect trust is employed, and it is based on the collective experience of peers, who have rated the trustworthiness of others [44]. Furthermore, it should take into account the social similarity between the IoT owners. Social similarity can be measured using a variety of approaches, including Cosine similarity and the Pearson correlation coefficient. Despite some criticism, these algorithms are widely used in social rating sites that rely on collaboration filtering to determine their rankings, which is why they are so popular.

### Direct trust computing

At any time, direct trust can be calculated by aggregating user *Ux* previous experiences with user *Uy* as follows [11, 19]:

$$di\_tr\left(Ux,Uy\right)=\frac{\propto \times \sum_{=1}^{m}pi\times \gamma^{\Delta ti}-\beta \times \left|\sum_{1}^{n}kj\times \delta^{\Delta tj}\right|}{\propto \times \sum_{=1}^{m}pi\times \gamma^{\Delta ti}+\beta \times \left|\sum_{1}^{n}kj\times \delta^{\Delta tj}\right|}$$ (1)

Where *m* and *n* represent the number of positive and negative self-experiences based on user *Ux* perception of user *Uy* respectively. *pi* and *kj* represent the quality of service or rate offered by user *Ux* to user *Uy*. ∝, *δ*, *γ* < 1, and β are ≥ 1. Both ∝ and β are weighting factors corresponding to positive and negative experiences where negative experiences are multiplied by a factor great than one since negative experiences are always with greater impact than positive ones. Δ*t* represents the time difference factor to overrule old values of *pi* and *kj*. We believe that direct trust between *Ux* and *Uy* is from the perspective of *Ux* not necessarily the same as that from the perspective of *Uy* which comes in line with [45]. This is in contrast to being friends on social media platforms such as Facebook, where if two users are friends, they appear on both of their friends’ lists. While on Twitter, for example, one can follow another and be added to his list, the person who is being followed is not required to follow the person who is following him back. In this study, we make the same assumption about trust and hence we can say that *tr*(*Ux*, *Uy*) ≠ *tr*(*Uy*, *Ux*).

As discussed earlier, some features are used in the assessment of trust values based on either of two approaches: direct trust and indirect trust. In direct trust, the user relies on his history of interactions using Eq (1). To minimise the error in the computation, and in case the user got engaged with Uy very recently and provided a new rank *tr*′, assuming both *tr*′ and *di_tr* are positive values, we can say that the mean square error (MSE) between all previous actual and directly calculated values (using Eq 1) between users Ux and Uy can be calculated as follows:

$$(MSE)=\frac{1}{n^{\prime }}\times \sum_{}{\left({tr^{'}}_{\left(x,y\right)}-{di\_tr}_{\left(x,y\right)}\right)}^{2}),$$

where *n*′ is the number of calculated points.

To keep *MSE* to a minimum, we can say that:

$$\frac{1}{n\prime }\sum_{}{\left({tr^{'}}_{\left(x,y\right)}-\frac{\propto \times \sum_{=1}^{m}pi\times \gamma^{\Delta ti}-\beta \times \left|\sum_{1}^{n}kj\times \delta^{\Delta tj}\right|}{\propto \times \sum_{=1}^{m}pi\times \gamma^{\Delta ti}+\beta \times \left|\sum_{1}^{n}kj\times \delta^{\Delta tj}\right|}\right)}^{2}=minimum.$$

For simplicity, we can say:

$$MSE=\frac{1}{n^{\prime }}\sum_{}{{(tr^{\prime \prime }}_{\left(x,y\right)}-\frac{\propto \times C_{1\left(x,y\right)}-\beta \times C_{2\left(x,y\right)}}{\propto \times C_{1\left(x,y\right)}+\beta \times C_{2\left(x,y\right)}})}^{2},$$

*where*

$$C_{1\left(x,y\right)}=\sum_{=1}^{m}pi\times \gamma^{\Delta ti},C_{2(x,y)}=\left|\sum_{1}^{n}kj\times \delta^{\Delta tj}\right|$$

Taking the derivative concerning β, we get:

$$\frac{2}{n\prime }\sum_{}{{(tr\prime }_{\left(x,y\right)}-\frac{\propto \times C_{1\left(x,y\right)}-\beta \times C_{2\left(x,y\right)}}{\propto \times C_{1\left(x,y\right)}+\beta \times C_{2\left(x,y\right)}})\times (0-\frac{-\left(\propto \times C_{1\left(x,y\right)}+\beta \times C_{2\left(x,y\right)}\right)\times C_{2\left(x,y\right)}-(\propto \times C_{1\left(x,y\right)}-\beta \times C_{2\left(x,y\right)})\times C_{2\left(x,y\right)}}{({\propto \times C_{1\left(x,y\right)}+\beta \times C_{2\left(x,y\right)})}^{2}})}^{}$$

we let the derivative equal zero and do further processing, we get:

$$\sum_{}{(tr\prime }_{\left(x,y\right)}-\frac{\propto \times C_{1\left(x,y\right)}-\beta \times C_{2\left(x,y\right)}}{\propto \times C_{1\left(x,y\right)}+\beta \times C_{2\left(x,y\right)}})\times (\frac{\left(\propto \times C_{1\left(x,y\right)}){\times C}_{2\left(x,y\right)}+\beta \times {{(C}_{2\left(x,y\right)}}^{2}\right)+\propto C_{1\left(x,y\right)}{\times C}_{2\left(x,y\right)}-\beta \times {{(C}_{2\left(x,y\right)})}^{2}}{({\propto \times C_{1\left(x,y\right)}+\beta \times C_{2\left(x,y\right)})}^{2}})=0,$$

then we can say:

$$\sum_{}\left({(tr\prime }_{\left(x,y\right)}-1\right)\times (\frac{\propto C_{1\left(x,y\right)}-\beta C_{2\left(x,y\right)}}{\propto C_{1\left(x,y\right)}+\beta C_{2\left(x,y\right)}})\times (\frac{2\propto C_{1\left(x,y\right)}{.C}_{2\left(x,y\right)}}{({\propto C_{1\left(x,y\right)}+\beta C_{2\left(x,y\right)})}^{2}})=0$$

Since *tr*′ and *di_tr* were assumed positive, then each summation item is positive. Hence, for the overall summation to be zero, each summation item should equal zero. Therefore, we get $\left({(tr\prime }_{\left(x,y\right)}-1\right)\times \left(\frac{\propto C_{1\left(x,y\right)}-\beta C_{2\left(x,y\right)}}{\propto C_{1\left(x,y\right)}+\beta C_{2\left(x,y\right)}}\right)\times \left(\frac{2\propto C_{1\left(x,y\right)}{.C}_{2\left(x,y\right)}}{({\propto C_{1\left(x,y\right)}+\beta C_{2\left(x,y\right)})}^{2}}\right)=0$. We can say that we have two cases to make MSE = 0: either (a) (*tr*′_(*x*,*y*)_ − 1) = 0, or (b) (∝ *C*_1(*x*,*y*)_ − *βC*_2(*x*,*y*)_) × (2 ∝ *C*_1(*x*,*y*)_ ⋅ *C*_2(*x*,*y*)_) = 0.

In the case of (a) and by substituting back in Eq (1) with *di_tr* = *tr*′ = 1, we get $2\beta \times \left|\sum_{1}^{n}kj\times \delta^{\Delta tj}\right|=0$. To achieve that, either *β* = 0 or $|\sum_{1}^{n}kj|=0$. This means that the mean square error approaches to a minimum if there are no negative experiences or their impact can be neglected. In case (b), by taking (∝ *C*_1(*x*,*y*)_ − *βC*_2(*x*,*y*)_) = 0 and substituting back in Eq (1), we get *di_tr* = 0, *tr*′ = 0 and $\beta =\left(\frac{C_{1}}{C_{2}}\right)\propto$.

From the above analysis, we can say that that *β* range of $\left[0,\frac{\left|\sum_{=1}^{m}pi\times \gamma^{\Delta ti}\right|}{\left|\sum_{1}^{n}kj\times \delta^{\Delta tj}\right|}\propto \right]$ will make *di_tr* range of [0, 1]. Further analysis is needed to allow continuous updating of alpha and beta values during run time according to each user case so that we can optimise the errors in the computation of direct trust compared to actual rates but this is considered out of the scope of this paper.

### Social similarity computing model

We assume that the influence of similarity of a trust feature (TF) should be considered in relation to the cardinality of the trustor’s trust feature only and not that of both the trustor and the trustee. In this context, we adjust the model of Jaccard [46] as follows:

$$Similarity(Ux,Uy)=\frac{\left|TF(Ux)\right|\cap \left|TF(Uy)\right|}{\left|TF(Ux)\right|},Similarity(Uy,Ux)=\frac{\left|TF(Uy)|\cap |TF(Ux)\right|}{\left|TF(Uy)\right|}$$

Where

Similarity(Ux,Uy)≠Similarity(Uy,Ux) (2)

Trust features are considered in calculating the social similarity index (SSI) associated with an IoT device owner as follows [12, 47]:

#### User centrality (Fr)

Each owner maintains a list of friends, which serves as a record of his or her social connections. The list of friends contains only direct friends of the owner of the IoT device, not friends of a friend. User centrality is based on the number of common friends a user has with others and through which we can measure his centrality to others. The higher the centrality of the user, the more convincing or influencing he becomes. User centrality or sometimes called friendship index (*Fr*) [12] can be measured by finding the ratio of the number of friends in common to the owner’s number of friends. The greater this ratio is, the higher the possibility that both communicating users share similar interests or views and consequently their devices.

$$Fr(Ux,Uy)=\frac{\left|Fr(Ux)\cap Fr(Uy)\right|}{\left|Fr(Ux)\right|}$$ (3)

Where |*Fr*(*Ux*) ∩ *Fr*(*Uy*)| is the list of common friends of users Ux and Uy respectively and |*Fr*(*Ux*)| is the number of friends the user Ux has.

#### Social Contact Index (Sc)

When two IoT devices, belonging to separate owners, are in the same place, their social contacts have a high likelihood of meeting up. Social interactions can include classmates, co-workers, and neighbours, among other people. The social contact index can be calculated as follows:

$$Sc(Ux,Uy)=\frac{\left|Sc(Ux)\cap Sc(Uy)\right|}{\left|Sc(Ux)\right|}$$ (4)

Where *Sc* (*Ux*) is the list of common social contacts visiting the same locations between users *Ux* and *Uy* respectively. *Sc* (*Ux*) and *Sc* (*Uy*) represent the total number of social contacts for *Ux* and *Uy* separately.

#### User Popularity (CoI)

Users belonging to a similar set of communities are likely to share mutual interests or skills. Community of interest could be measured in the same way as follows:

$$CoI(Ux,Uy)=\frac{\left|CoI(Ux)\cap CoI(Uy)\right|}{\left|CoI(Ux)\right|}$$ (5)

|*CoI*(*Ux*) ∩ *CoI*(*Uy*)| is the list of common communities, while *CoI* (*Ux*) and *CoI* (*Uy*) are all communities both users *Ux* and *Uy* are members of. Here we refer to *CoI* as how popular or public the user is compared to others concerning membership in community groups of interests. The more subscribed the user to communities, the more he becomes public and popular, and therefore we refer to this feature as user popularity.

#### IoT device Proximity (Prox)

Here IoT devices can come across each other, and if a device interrogates another device, we say that the device comes into proximity. Proximity can be foreseen using Bluetooth or any other wireless communication protocol. Additionally, it can be identified using GPS location services. Proximity is rather asymmetric which means that proximity occurrences where user *Uy* devices get seen by user *Ux* devices do not necessarily equal those of user *Ux* devices seen by user *Uy*. User proximity is calculated as follows:

$$Prox(Ux,Uy)=\frac{\#of\;times\;Uy\;comes\;in\;proximity\;with\;Ux}{\#of\;times\;all\;users\;come\;in\;proximity\;with\;Ux}$$ (6)

#### Interaction & Reliability (Cc)

Cc measures how often a user has been contacted by other users or been in contact with others. User Interaction is computed by messages or communication events with other users:

$$Cc=\frac{\#of\;interctions\;between\;Ux\;and\;Uy}{\#of\;interactions\;between\;Ux\;and\;all\;users}$$ (7)

#### Social Similarity (SSI)

The social similarity index (SSI) between two IoT devices owners *Ux* and *Uy* could be calculated as follows:

$$SSI\left(Ux,Uy\right)=\omega_{1}.Fr\left(Ux,Uy\right)+\omega_{2}.Sc\left(Ux,Uy\right)+\omega_{3}.CoI\left(Ux,Uy\right)+\omega_{4}.Prox\left(Ux,Uy\right)+\omega_{5}.Cc(Ux,Uy),$$

Where

$$\sum_{i=1}^{5}w_{i}=1$$ (8)

### Indirect trust calculation

In case a user cannot judge other users’ trustworthiness on his own, he or she can make use of other users’ ratings. Here we use social similarity to weigh other users’ direct trusts so to degrade malicious users’ ratings and appraise those by close social contacts. Thus, we can compute indirect trust between users *Ux* and *Uy* as follows:

$$in\_tr=\frac{1}{m}\times \left(\sum_{i=1}^{m}SSI\left(Ux,Ui\right).tr\left(Ui,Uy\right)\right),$$ (9)

where *m* is the number of users.

### Highly trusted users (TP)

We define highly trusted users to be users scoring the highest records in any of the following:

- Quality of service offered to users by the user or user offering a service or information
- User centrality
- User popularity
- Social contact
- IoT device Proximity
- interaction and reliability
- Average Social Similarity index

A list TP is updated periodically with top users on the above features, and their evaluation is being done on a global basis. In [11], two factors were used to weigh trust ratings; instead, we use aggregated social similarity index (SSI) between a user *Ux* and a user *Utp* ∈ *TP* as a measure of trust that user *Ux* has in user *Ut*_*p*_ rating as follows:

$$tp\_tr(Ux,Uy)=\frac{1}{k}*\sum_{j=1}^{k}SSI(Ux,Utpj)\times tr(Utpj,Uy)$$ (10)

### Trust aggregation

**Fig 1:**
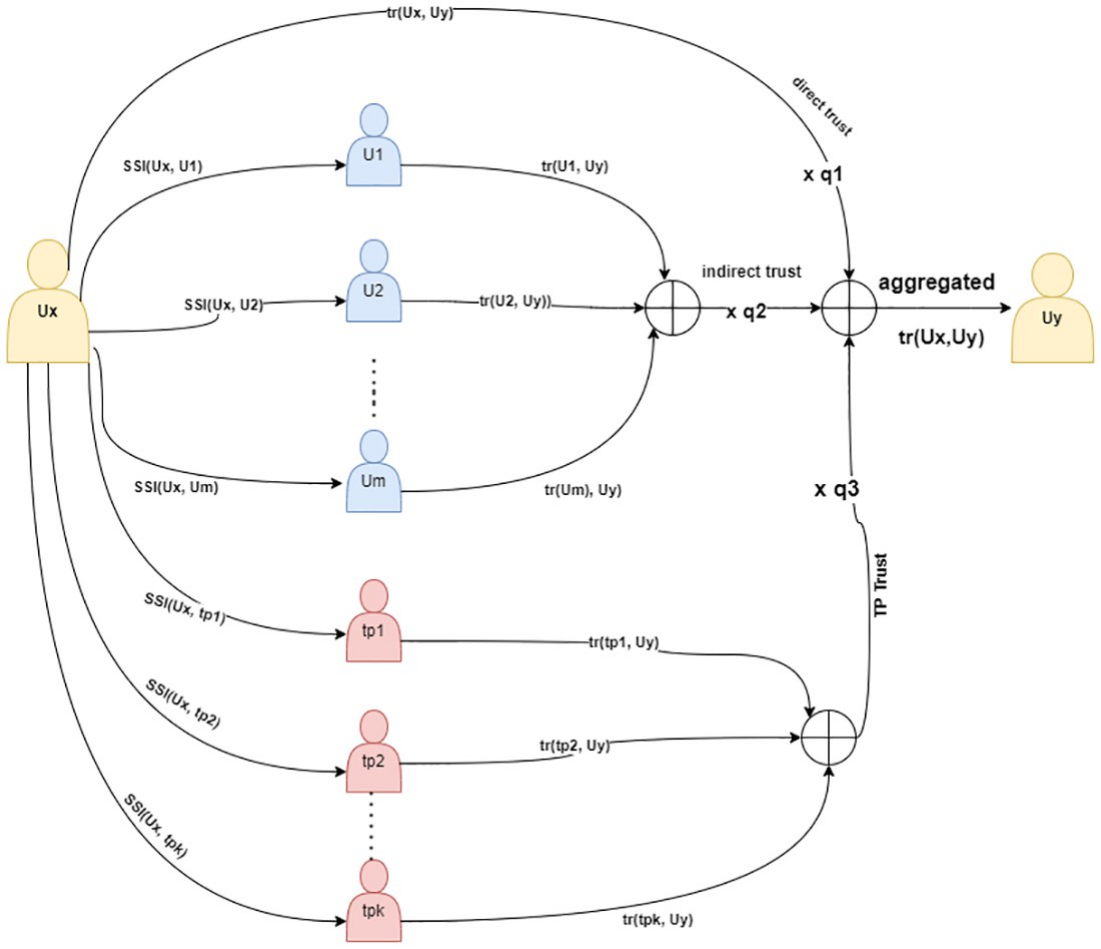
Visualization of the proposed aggregation of computed trust.

To calculate an overall trust value which *Ux* has on Uy, we need to aggregate calculated trust as follows (see Fig 1):

$$\text{tr}\left(\text{Ux},\text{Uy}\right)=q_{1}.\left(di\_tr\right)+q_{2}.\left(in\_tr\right)+q_{3}.\left(tp\_tr\right),$$

Where *q*_1_ = 1 − (*q*_2_ + *q*_3_), *q*_1_, *q*_2_, *q*_3_ < 1 assuming *q*_2_ = *q*_3_ = 1 − *q*, then we can say that:

$$tr\left(Ux,Uy\right)=q.\left(di\_tr\right)+(1-q).(in\_tr+tp\_tr)$$ (11)

At a given time and based on the value of *q*, we can control the contribution of direct trust, indirect trust, and highly trusted users in *Ux* final perception of *Uy*. From Eq (11), we can see that the aggregated trust value depends mainly on combining the directly computed value which represents *Ux* perception of *Uy* directly as shown by Eq (1) and trust values provided by others i.e. other users and highly trusted users. In this work, to execute Eq (11) concerning the value of *q*, we can divide users into four groups based on their attitudes toward data privacy and sharing information on social networks:

#### Less social users (q ∈ [0.4, 0.7])

In this profile, a user does trust his assessment only based on his or her history of interactions with the trustee user and gives a low priority to his socially similar contacts. This one requires previous experience with service providers so that the model can converge. Thus, the aim will be to find a local and recent direct trust value using Eq (1). This case can be beneficial in the case of payment service providers, restaurants services, or other highly confidential services.

#### Average social users (q ∈ [0, 0.4])

Like highly strict users, this category of users gives high priority to their assessments but at the same time takes into consideration social similarity factors but with less priority.

#### Highly social users (q ∈ [0, 0.4])

This category has a very large trusted social network and can safely rely on their assessments.

#### Adaptive trust aggregation (dynamic q)

Based on users’ previous interactions with other users, we can adapt the value of *q*. To find the optimal value of q, we assume that the most recent given rank by *Ux* to *Uy* is *tr’* such that mean square error, $MSE=\frac{1}{n}\sum_{y}{\left((tr^{\prime }-(q.di\_tr+\left(1-q\right).\left(in_{tr}+tp\_tr\right))\right)}^{2}$, taking the derivative concerning *q*, we get:

$$\frac{dMSE}{dq}=\frac{2}{n}\sum_{y}\left((tr^{\prime }-{in_{tr}-tp\_tr)+q(di_{tr}-in\_tr-tp\_tr}_{}\right))\times (di_{tr}-in\_tr-tp\_tr)$$

$$=\frac{2}{n}\sum_{y}\left((tr^{\prime }-{in_{tr}-tp\_tr){+q.(di_{tr}.}_{}-in\_tr-tp\_tr}_{}\right))\times (di_{tr}-in\_tr-tp\_tr)$$

To find the minimum value of MSE, we let $\frac{dMSE}{dq}=0$, then we get:

$$\sum_{y}((tr^{\prime }-{in_{tr}-tp\_tr){\times (di_{tr}-in\_tr-tp\_tr)=}_{}}_{}-q\times \sum_{}\left(di_{tr}-in\_tr-tp\_tr\right)\times (di_{tr}-in\_tr-tp\_tr)$$

Then,$q\prime (optimal)=\frac{-\sum_{y}(tr^{\prime }-{(in_{tr}+tp\_tr)){\times (di_{tr}-(in_{tr}+tp\_tr))}_{}}_{}}{\sum_{y}{\left(di_{tr}-in\_tr-tp\_tr\right)}^{2}}$

This means that at any given time, knowing the rank or quality of service users give to others, we can calculate *q* from Eq (13) as follows:

q=q´=-∑y(tr′-(intr+tp_tr))×(ditr-(intr+tp_tr))∑y(ditr-in_tr-tp_tr)2,q′<11,q′≥1 (12)

From values of {*in*_*tr*, *tp*_*tr*, *tr*′, *di*_*tr*} per user *Ux* as trustor with other users *Uy* as trustees, optimal values of *q* can be obtained using formula (12) at any given time for that particulare user.

### Dealing with attacks

Network operations can be disrupted by a malicious user launching security attacks. Due to this assumption, such an effort will be addressed by Intrusion Detection Systems and is therefore out of the scope of this study. In this research, we study attacks on the trust management system that aim to increase or harm users’ reputations. Several attacks target systems that handle trust and reputation. It is feasible for groups of people to launch offensive operations on trust management systems. Among the many examples is bad-mouthing [48]. Attackers coordinate to generate bad feedback on the target, tarnish or undermine its reputation. By conflating trust and reputation, collusive activity may result in the blocking of valid paths in the network [49]. In ballot stuffing attacks, a group of entities agrees to provide fake positive feedback on another, resulting in the entity swiftly earning a high reputation. Collusive users’ goal is to deceive the trust mechanism and cause it to fail to appropriately reflect the trustworthiness of an assessed user [49]. Opportunistic Attacks represent other types of attacks where malicious users behave well and poorly alternately in an on-off attack to remain unnoticed while causing damage [50]. Unfortunately, as their trust value increases, these rogue users may suddenly launch attacks. This changing behaviour makes it difficult to detect such malicious users. It provides good services or ratings when its reputation drops to improve and when it becomes accepted, it can perform malicious again doing bad-mouthing or ballot-stuffing [1]. In self-promoting attacks, a malicious user can provide itself with a good rating to be seen with a higher reputation.

Our proposed model helps deal with badmouthing, ballot stuffing, and self-promoting attacks mentioned above as the highest trusted parties are updated continuously and can help filter such attacks. Social similarity helps as a weighting factor of user recommendations so to increase the impact of those having high social similarities with users and reduce the impact of malicious or unknown users.

## Proposed trust computing architecture

**Fig 2:**
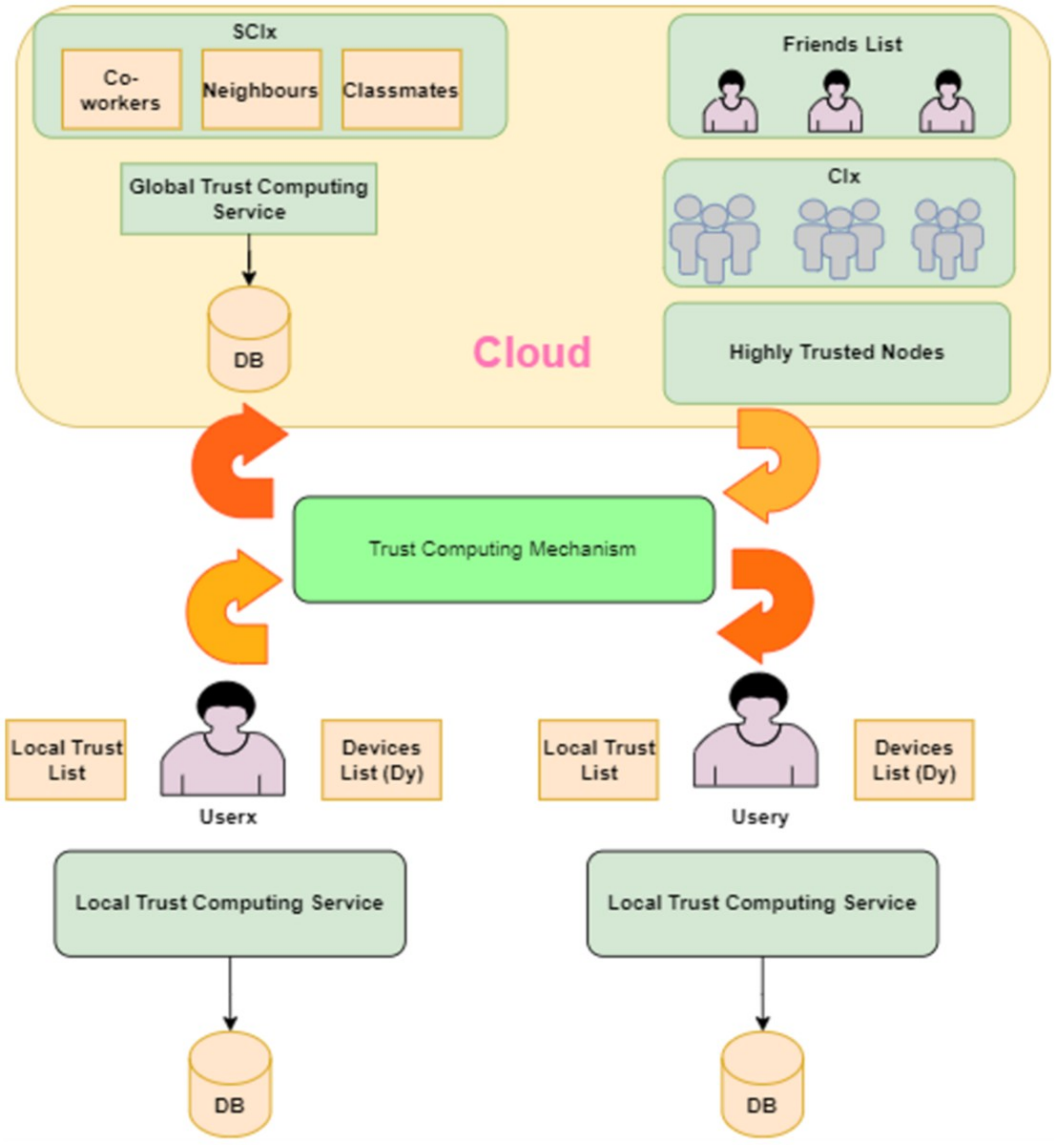
Trust computing high-level functional architecture.

**Fig 3:**
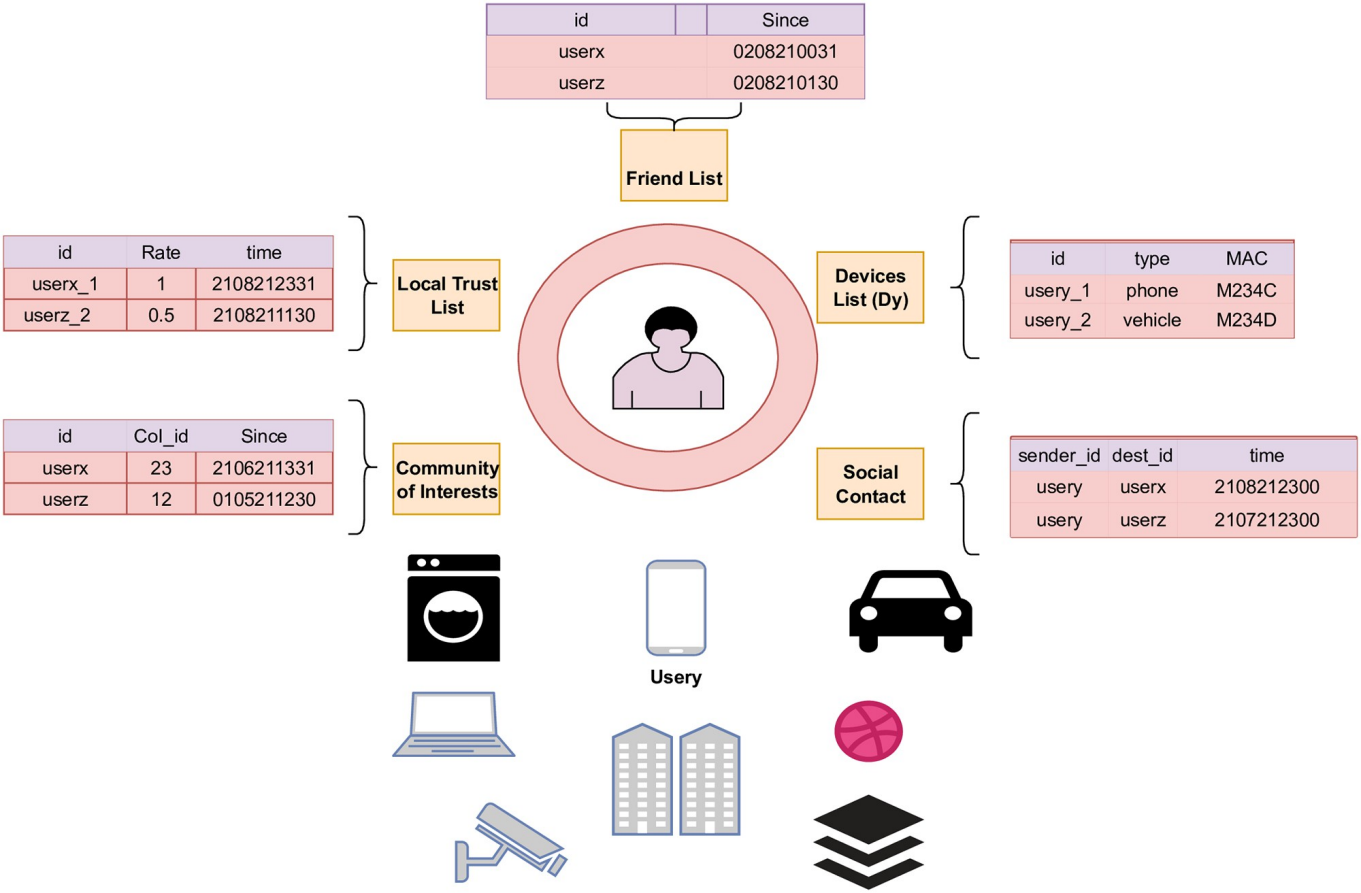
Local trust computing architecture.

Fig 2 shows the high-level architectural design of the proposed model. A local trust computing service calculates trust based on the computing Eqs (1–12). A global trust computing service calculates trust based on data stored on the cloud using a machine learning approach. Fig 3 illustrates the many lists maintained on the user’s side to assist him in making an informed rating judgment about other users. Each user ranks his or her experience with another user in the Local Trust Table for each communication. If contact is initiated with other users, the social contact table is updated. All conducted interactions with other users are stored locally including locations, messages, and devices proximity occurrences.

Membership in a user community of interest is stored in the user’s community of interest table. The friend list table stores information about friends.

To link the user to his IoT devices, a smartphone owned by the user must run the local trust service that allows the user to control his IoT devices, manage locally stored data, communicate with other users, and make a trust calculation. Additionally, it connects to the global trust computing service as will be discussed later.

### Local trust computing service

If a user contacts another user or wishes to register for a service provided by a certain service provider, a local service operating on his smartphone is triggered which does the following activities: -

- searches for the service provider’s trust features in the user’s local trust table.
- Collect friends’ ratings of that trustee. This is done by calling a function to find rates provided by user Ux’s friends stored globally within a predefined time frame and based on the user profile with the following options:
  - Get friends rates only
  - Include all social similar users
- Continuous updates sent to global database tables

If the service provider’s or trustee cannot be computed due to missing trusted rates of that user, a call is made to a global trust service running on the cloud server to provide a recommendation using a machine learning model based on a KNN classifier which will be described in the next sections.

### Global trust computing service

**Fig 4:**
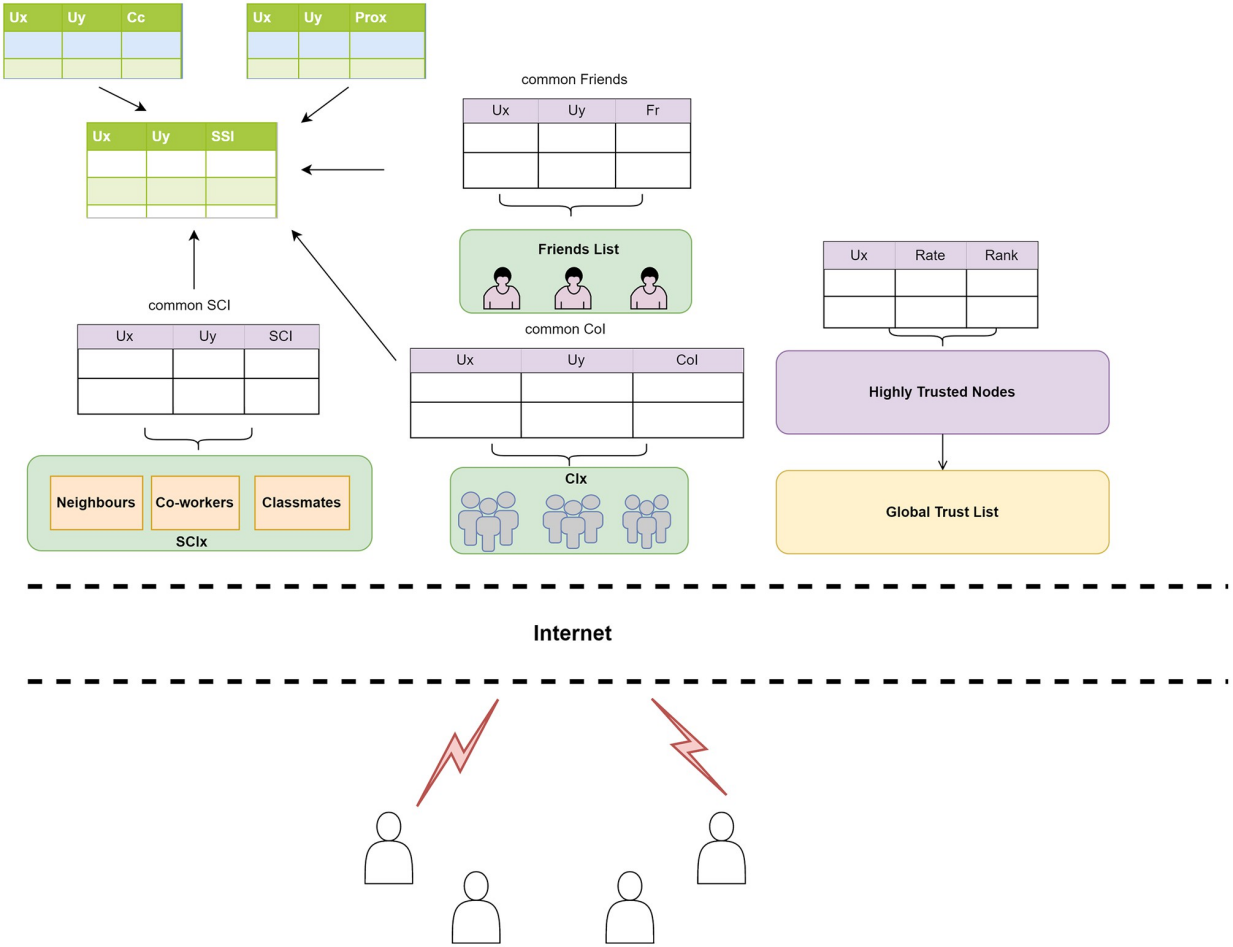
Cloud-based trust computing architecture.

As previously said, not all users can determine the trustworthiness of other users locally; so, a cloud-based approach is applied in this study. All data acquired locally on the user side is uploaded to the cloud and stored in the global database tables. Each mobile device keeps track of all performed transactions on the local database using a logging mechanism. This transaction log is uploaded to the cloud on predefined periods allowing for uploading only changes made and not the whole database. The architecture is depicted in Fig 4. Common tables are produced and maintained based on regularly submitted data from users’ local tables. Furthermore, whenever a user’s transaction log is executed on the cloud, trust tables are updated in the cloud. As a result, the cloud data reflects the most recent values from local tables with an acceptable time delay.

Below are the functionalities provided by the global trust computing service:

#### Extracting and calculating social similarity features

At the cloud, friends are registered on the run as users get new ones. As such, a global friends table is maintained on the cloud side. Each time a user adds/removes a friend, the update is synchronized from the user’s client app to the cloud via the local trust service. Based on this global friend table, a common friends table can be created and updated. Similarly, in the database, common social relationships and communities of interest can be located and preserved independently. It is feasible to count the number of mutual friends / social contacts/community of interests and remaining features between two users: user U*x* and user U*y* at any given moment and hence social similarity index (SSI) can be calculated and stored on the cloud.

#### Applying a machine learning model for trust prediction

As users use the model, daily interactions are stored in the system with all features as mentioned earlier. The main purpose for a user U*x* who does not know user U*y* is to predict *tr*(*Ux*, *Uy*) from the user’s previous interactions. The system records all interactions made and extract the features below from the data: user centrality (*Fr*), Social Contact Index (Sc), Community of Interest or user popularity (CoI), Interaction and reliability (Cc), Proximity (Prox), and quality of service (Y). Before starting the clustering approach, outliers need to be removed as they can lead to misleading results. An outlier has a value that is more than three scaled median absolute deviations (MAD) away from the median [51]. Therefore, they are removed from the data as a pre-processing step. A clustering approach using the K-means algorithm is applied to classify the data into clusters. The K-means algorithm divides data into k equal-variance clusters where the number of classes is chosen by the user. Feature scaling is necessary to avoid domination of some features like IoT device proximity and users’ interaction compared to features like user similarity and quality of service which are in the range of [0, 1]. Thus, we choose to normalize the features using min-max normalization which rescale features to the range of [0, 1] to help deal with this issue using the following formula:

$$x^{\prime }=\frac{x-min(x)}{\text{max}\left(x\right)-min(x)}$$ (13)

**Fig 5:**
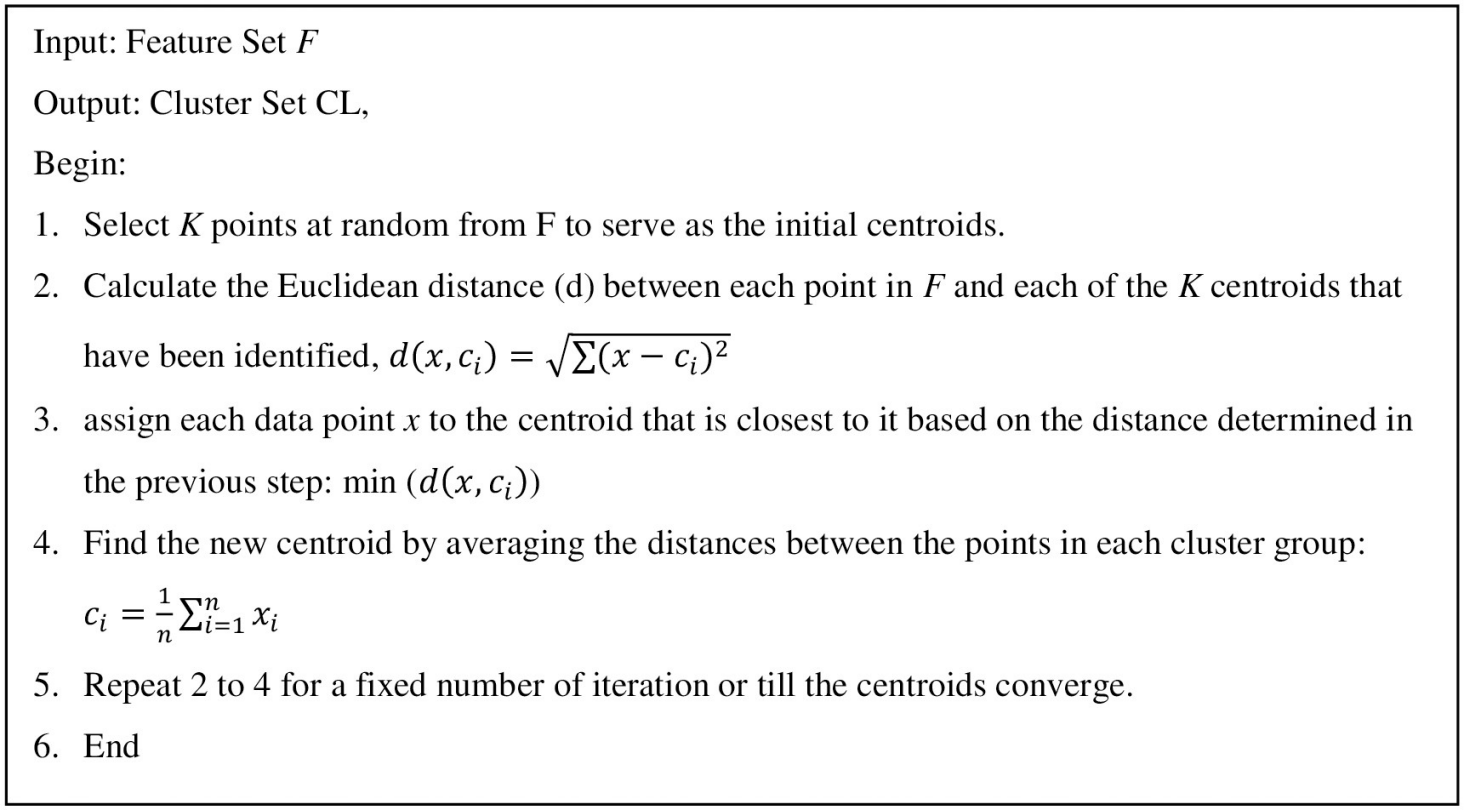
Clustering algorithm (Algorithm I).

K-means clustering (with k = 2) is used to partition observations into k clusters that are mutually exclusive and as near to each other as possible. The algorithm steps are shown in Fig 5. Each cluster has a unique centroid. Despite its speed and simplicity in comparison to other approaches such as k-medoids, one disadvantage of k-means is that it does not account for outliers. As mentioned earlier, and as a pre-processing step, we remove outliers from the data using the median absolute deviations (MAD) [51].

**Fig 6:**
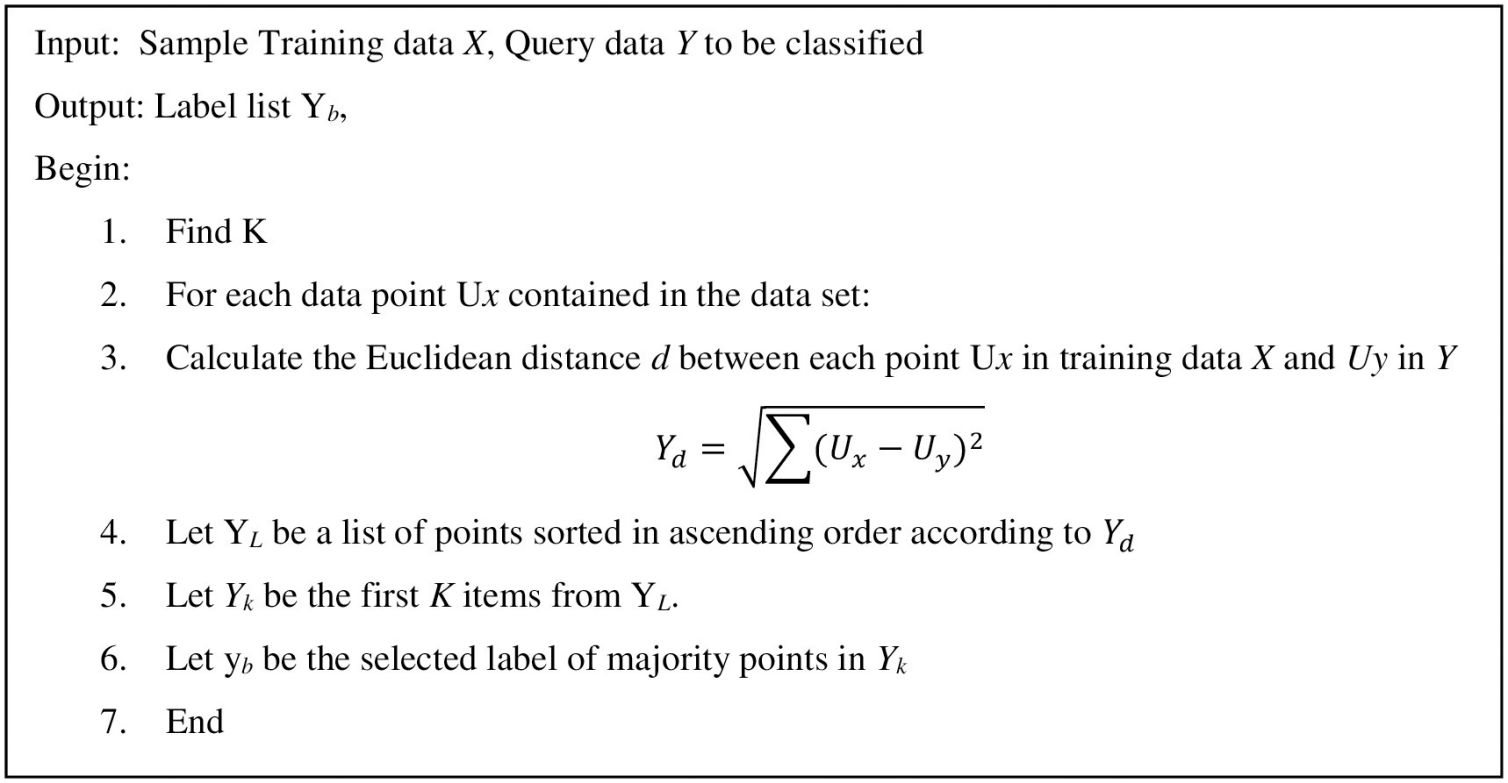
Classification algorithm (Algorithm II).

After the k-means algorithm converges, it can be used for classification with a small number of labelled examples. By knowing a sample of observations labels residing in each cluster, we label those clusters as trusted (tr = 1) / untrusted (tr = 0). When getting new sample data, we simply find the nearest neighbour point using the k-nearest neighbour (KNN) algorithm to find which cluster it belongs to. Knowing that cluster label, we can label the new point/sample. Fig 6 shows the steps of the classification algorithm. For empirical work, we will divide our dataset into training (80%) and testing (20%) with cross-validation to achieve more accurate results.

## Empirical work

We used 40 cores—64GB RAM Dell R740 Server with MATLAB R2021a to do the computations. For the sake of simplicity, all computations ran from the server where data are located. A dataset compiled by the social mobile application MobiClique was used to simulate our proposed models [52]. The proposed trust computing model Eqs (1–13) and the machine learning model were applied. Result data produced in this work are available in S1 File. In this experimental work, we assumed that people already on the friend list were trusted. Moreover, we assumed first contact profile exchange between users as positive trust (tr = 1) while those with no profile exchange as distrust (tr = 0). Besides, we extracted people who came in first contact and exchanged profiles. From which we found those who exchanged messages back and forth as trusting each other initially. The user that sends a contact message to another user, initially is trusting that user and vice versa. Since the dataset was collected during a conference, all users were noticed to be in the same physical neighbourhood thus we assumed ScI = 1 and hence was not considered as an influencing factor. Further, we assume ∝ = β = 1, with no time decay for Eq (1) and concerning Eq (9), *ω*_1_ = *ω*_3_ = 0.4, *ω*_4_ = *ω*_5_ = 0.1.

The used dataset consists of 76 users with 76x76 (5776) observations. All features were extracted from the dataset in the form: (Ux, Uy) value. Missing values were assumed to be zero. All values were aggregated in one big matrix called Feature with size 5776 x 11 where columns represent the features and rows represent the observations. The first five columns represent the input features while the remaining six columns are the different computed trust values respectively; direct trust (*di_tr*), proposed indirect with social similarity (*in_tr*), TP trust only (*tp_tr*), traditional indirect trust (without social similarity), proposed aggregated (*tr*) and traditionally aggregated. In the learning model, we used the first five columns as input to the model while the outcome was compared to the actual values after mapping them to [0, 1] such that values below 0.5 are considered untrusted (tr = 0) while values greater or equal 0,5 are considered trusted (tr = 1). We applied our ML model to verify found trust relationships.

**Fig 7:**
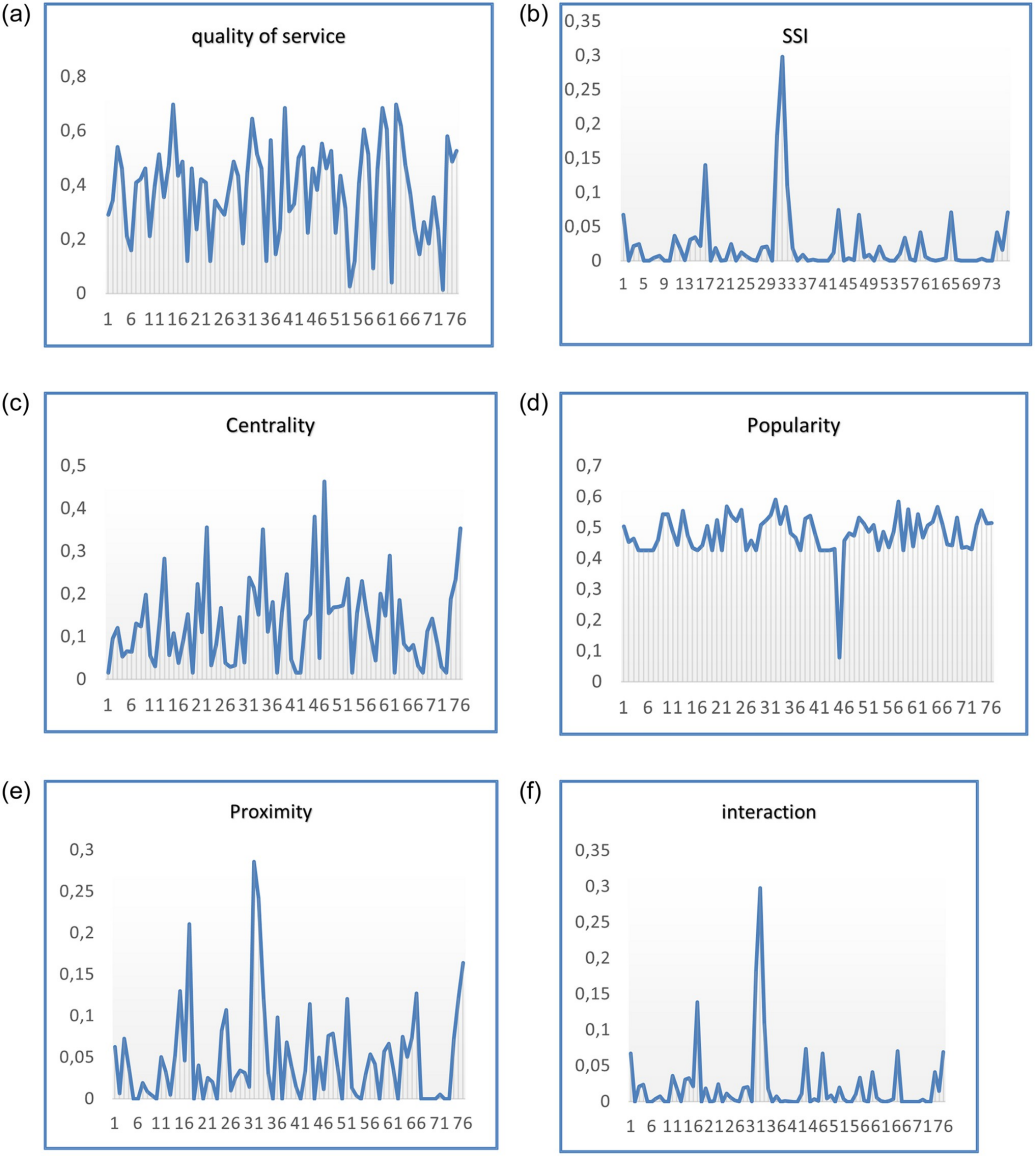
Overview of obtained trust features values per users in the dataset. (a) U63 with the highest rate, (b) U32 as most social, (c) U47 as most central, (d) U32 as most popular, (e) U31 as highest in proximity, (f) U32 as most interactive.

Fig 7 shows the distribution of the values of trust features among the users. The obtained results showed that the highest in quality of service users was “user 63” (see Fig 7a), most central was “user 47” (see Fig 7c), most social, popular and interactive was “user 32” (see Fig 7b, 7d and 7f), and the one with the highest proximity as “user 31” (see Fig 7e).

**Fig 8:**
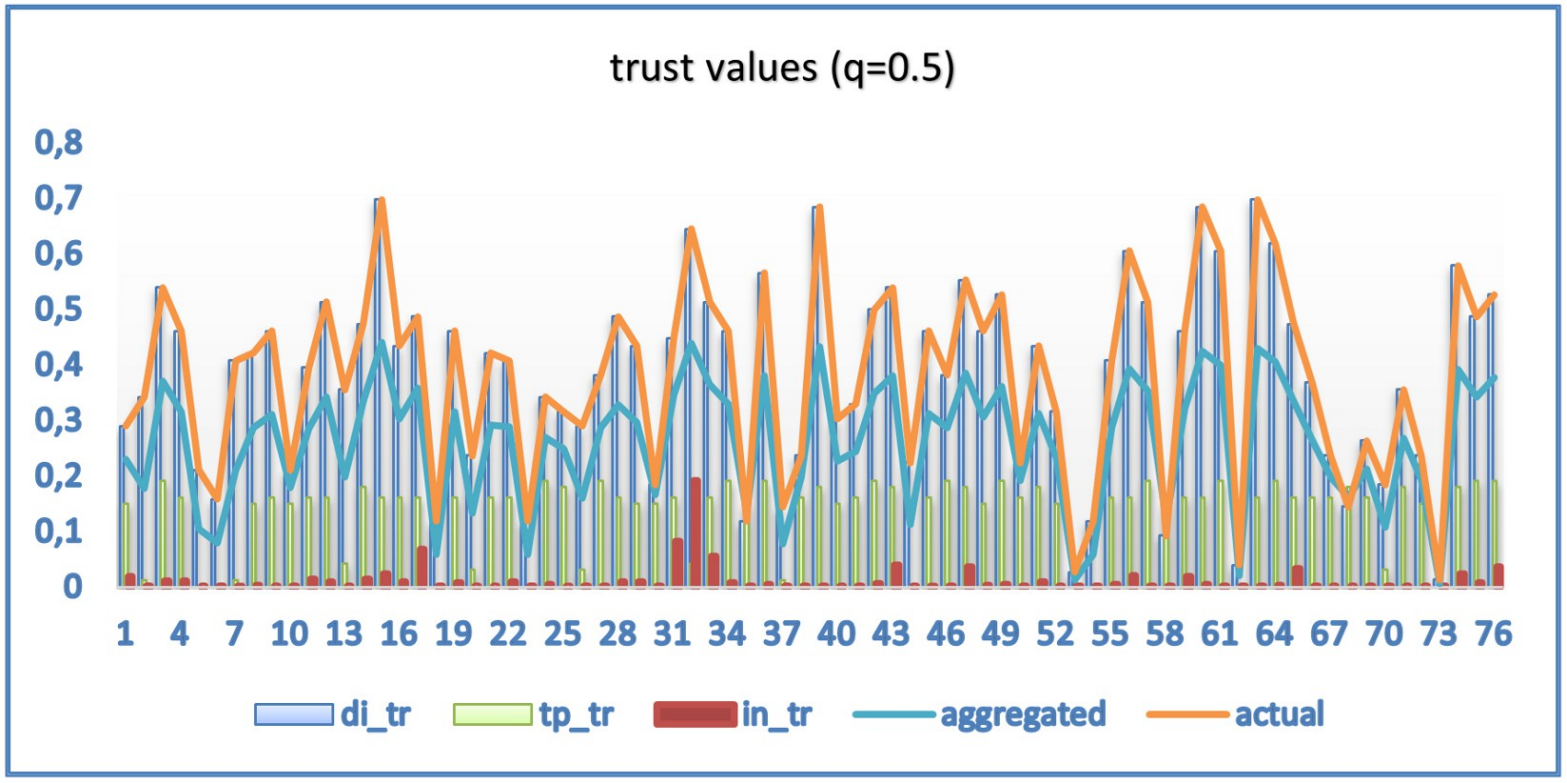
Averaged user trust distribution (q = 0.5).

**Fig 9:**
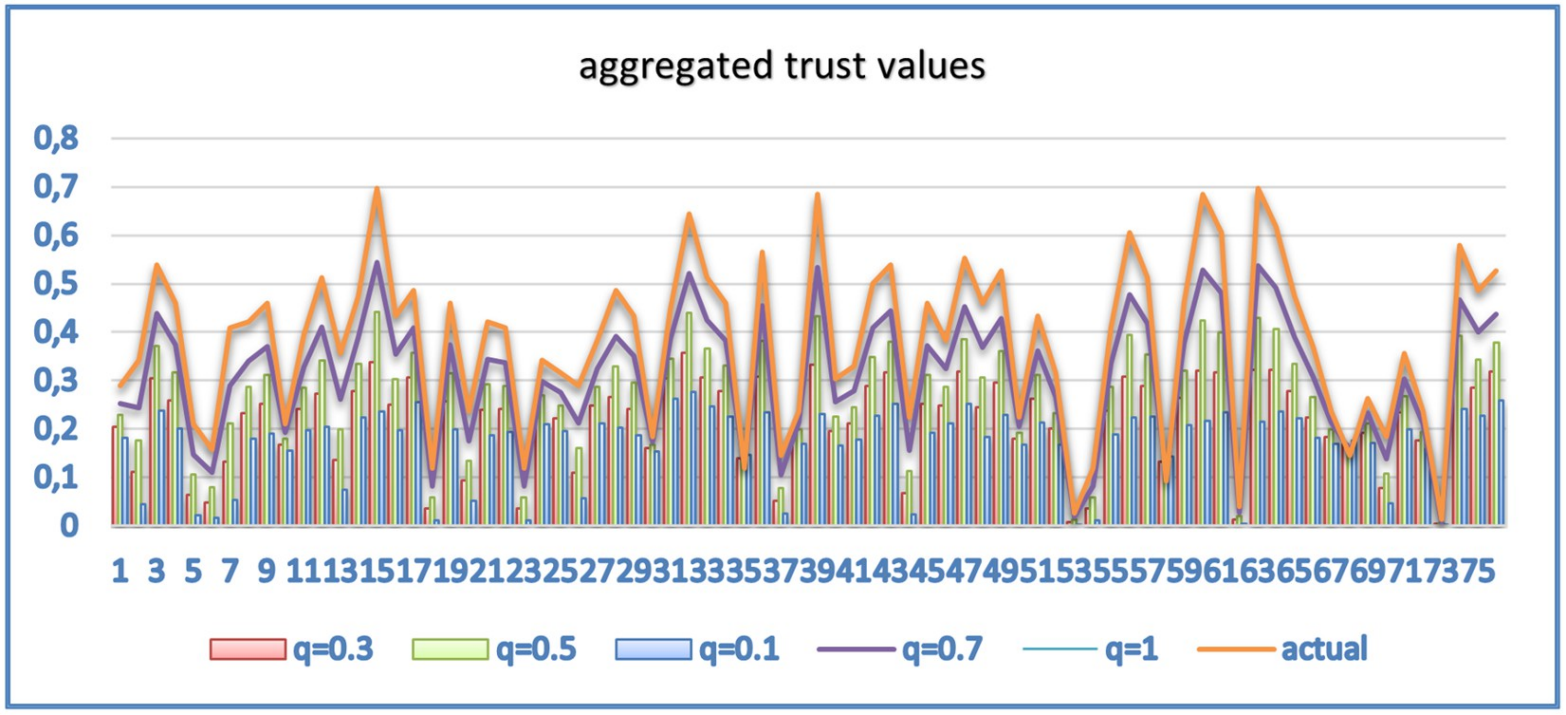
Aggregated trust values for different q values.

Fig 8 shows the averaged computed trust distribution per user concerning direct trust (di_tr), indirect trust (in_tr), highly trusted users trust (tp_tr), aggregated trust and actual values. The value of *q* determines the contribution of direct, indirect, and tp trust to the overall computed value. As discussed earlier, changing *q* values in Eq (11) will impact the contribution of direct, indirect, and trusted parties to the overall trust a user U*x* has on user Uy. Fig 9 shows the results corresponding to different static values of q. Other weighting factors, used in the computation, could have an impact on the accuracy of the model and thus need to be further studied for an optimal combination [12]. In this experimental work, indirect trust was not dominant because it was influenced by users’ social similarity with other users, which was not all that high.

**Fig 10:**
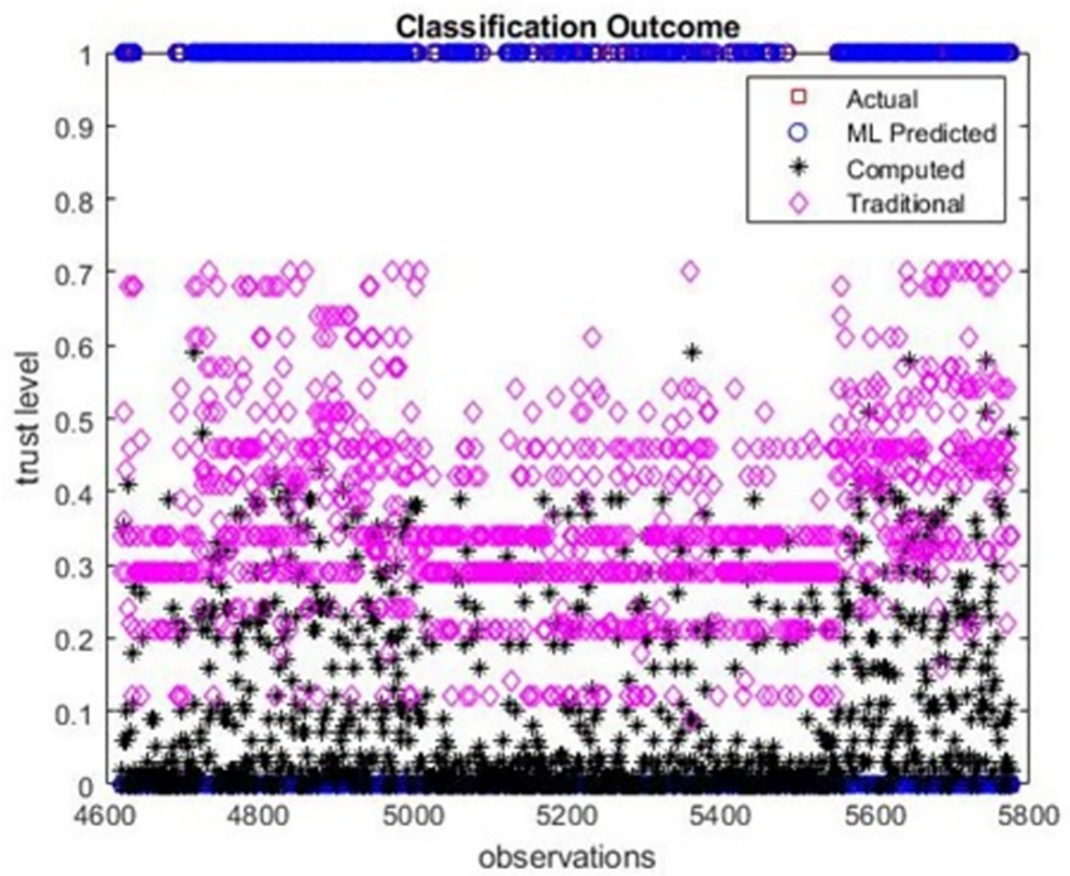
Classification results for testing data compared to computed and traditional approaches (q = 0.5).

As expected, the use of machine learning has helped in predicting trust more accurately. In this work, we implemented algorithms (I) and (II) on the extracted features from the dataset. Fig 10 shows actual versus those predicted by machine learning (ML Predicted), proposed computational (computed by formulas 1–10), and the traditional method. A threshold of 0.5 is used such that trust levels above or equal ‘0.5’ are considered trusted (tr = 1) and those below ‘0.5’ are considered untrusted (tr = 0). Traditional and computed values are somewhere between ‘0’ and ‘1’ as seen in the figure and are classified into two separate classes of data observations: trusted (tr> = 0.5) and untrusted (tr<0.5).

## Proposed models performance

In this paper, a hybrid approach is proposed for trust computing based on two models; one is based on a distributed computational model using social similarity and previous interactions and the other one is based on a global and a centralised machine learning model. In the following, the performance of the proposed approaches is validated concerning trust prediction and resilience to attacks.

## Trust prediction performance

Table 1 shows the performance of the proposed approaches. The proposed machine learning model has classified all classes correctly with a precision of 100%. The proposed computing model (Eq 11) performance scored 100% however we expect the performance to depend on the choice of the weighting factors as mentioned before. The social similarity was noticed to highly impact the trust levels of users. This could be due to the nature of the dataset as it was collected in a few days not giving users enough time to socialize together and build up new friendships and social contacts.

**Table 1:** Confusion matrix of the proposed models compared to the traditional method.

	Trusted	Untrusted	Precision	Recall	Accuracy
**Proposed ML**	TP = 749	FN = 0	1	1	1
	FP = 0	TN = 407			
**Aggregated computed (q = 0.5)**	TP = 749	FN = 0	1	1	1
	FP = 0	TN = 407			
**Traditional**	TP = 749	FN = 0	1	1	1
	FP = 0	TN = 407			
**Direct Trust (di_tr)**	TP = 749	FN = 0	1	1	1
	FP = 0	TN = 407			
**Indirect Trust (In_tr)**	TP = 749	FN = 0	0.65	1	0.65
	FP = 407	TN = 0			
**Highly Trusted Parties (tp_tr)**	TP = 749	FN = 0	0.65	1	0.65
	FP = 401	TN = 6			

**Fig 11:**
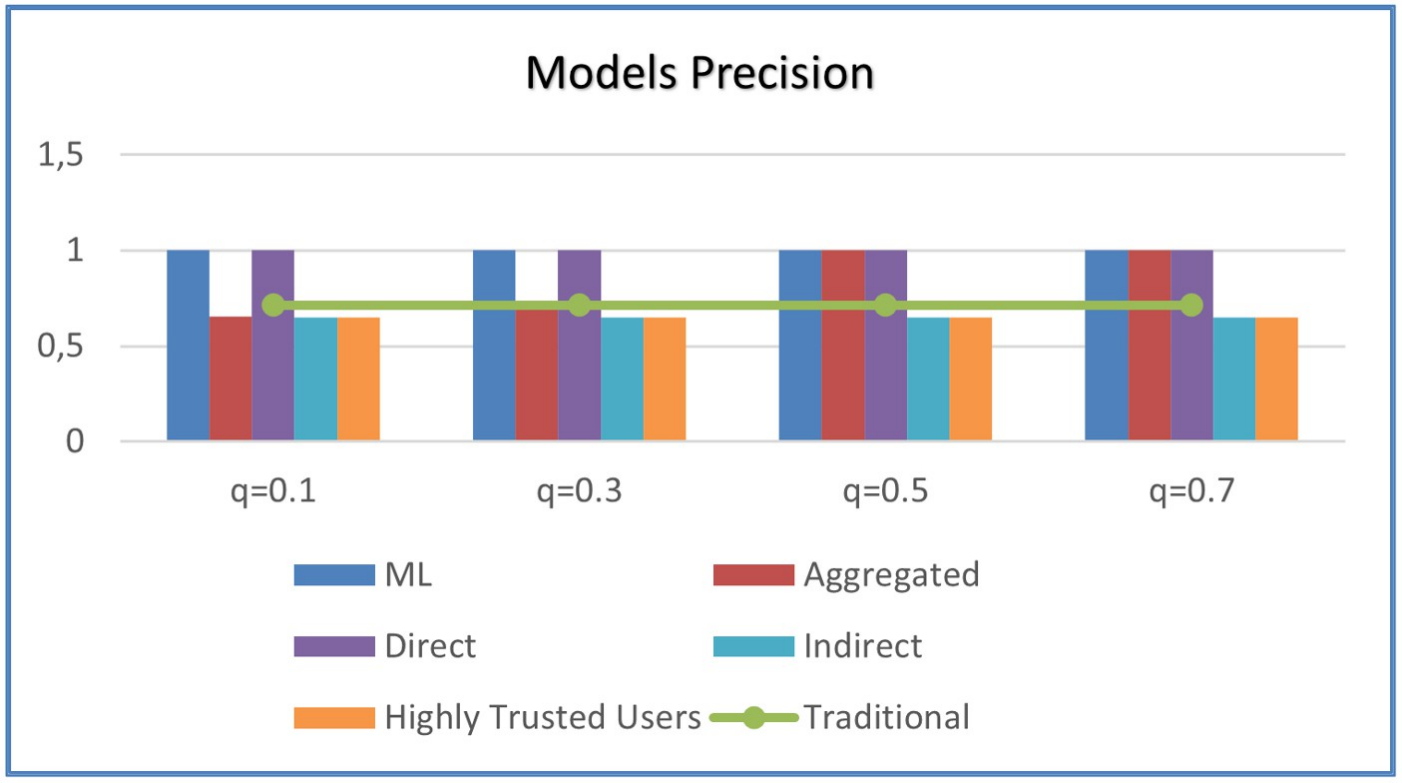
Effect of aggregation weighting factor (q) on prediction precision.

We notice also that trust computed with the help of peers is less accurate compared to the direct one which is logical as the indirect trust does not necessarily have to meet with users self-perception. Therefore, we propose an aggregation of the three computed values to maximize the benefit. To do that, we need to use an optimal value for *q*. Dynamic *q* calculation aids in determining a value for *q* that brings the aggregated value close to the user’s most recent actual trust value. Dynamic trust aggregation was obtained by using Eq (12) in calculating optimal values of *q* per user and applying that in the aggregation formula of (11). Using the formula (12), we can obtain optimal values of *q*. In this experimental work, we notice that *q* values are “1” which is due to that there were no changing users’ interactions over different periods detected in the dataset. However, when using the system in real-time, it is expected that we capture different interactions over different periods and thus different *q* values will be calculated per user. Fig 11 shows the performance when using different global values of *q* in Eq (11). It is noticeable that the proposed ML was not affected by statically changing *q* values.

**Fig 12:**
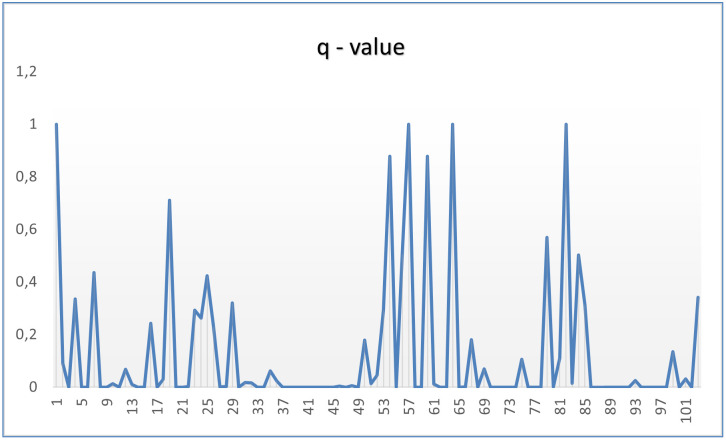
Obtaining dynamics in q values when using a dynamic dataset called Epinion.

To further test Eq (12) on a rather dynamic dataset, we chose the Epinion dataset [53] which has several previous interactions between the same users and can show the changing values of *q*. To do that we, extracted interactions between 103 users with 37 others such that our feature matrix contained 3811 (103x37) observations where friendship similarity was the only social feature. Obtained different values for q for users are shown in Fig 12.

**Fig 13:**
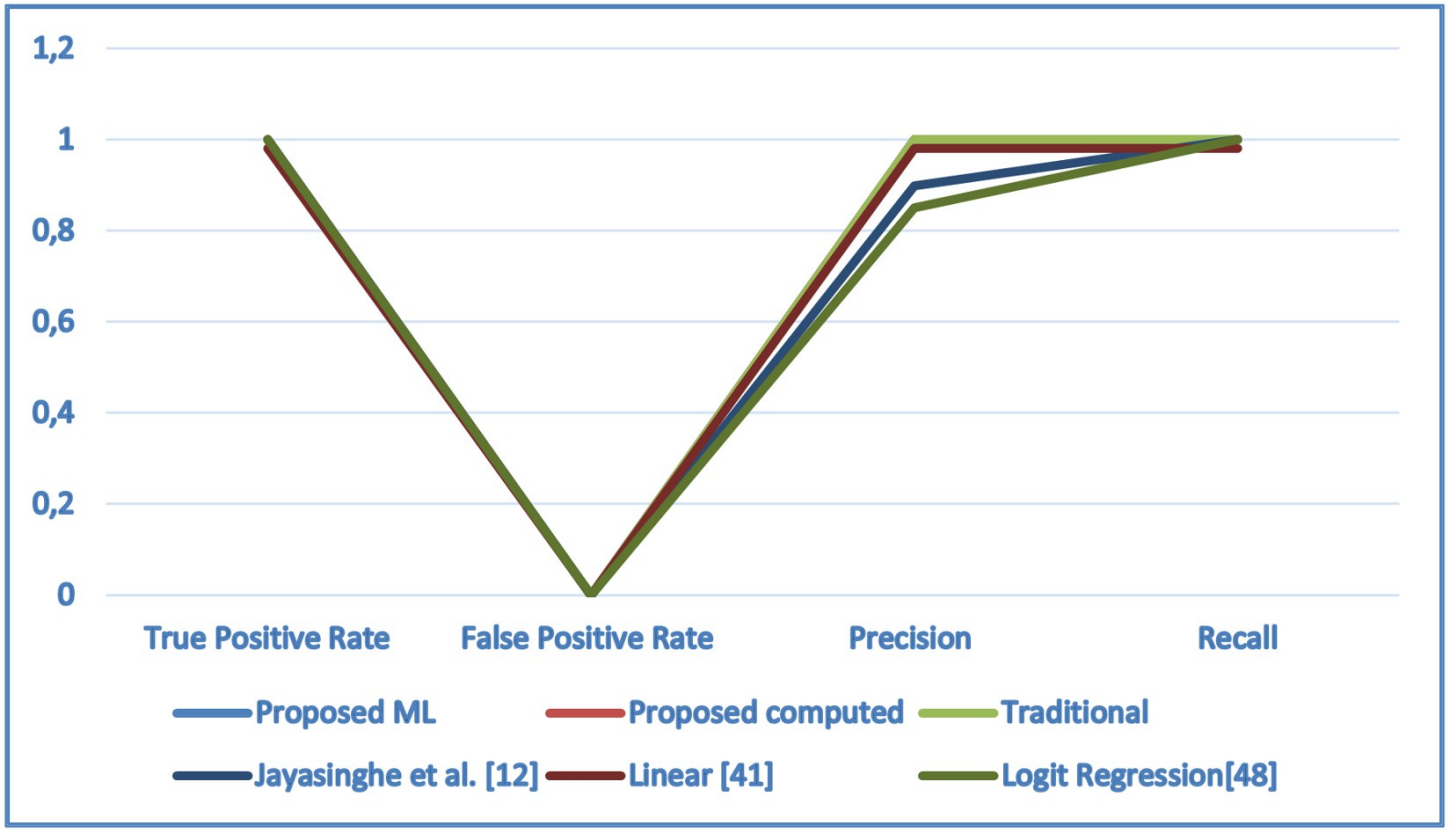
Comparison of proposed approaches to related work.

Fig 13 shows a comparison between trust computed using machine learning, the proposed social-based computing model, the traditional approach, and the work of Jayasinghe et al. [12], the Linear approach of [43] and the logit Regression approach of [54] which used the same dataset. As can be seen, the performance of the approaches is close concerning false positive and true positive rates in general but the proposed approaches were more accurate (better in precision and recall).

### Resilience to attacks

**Fig 14:**
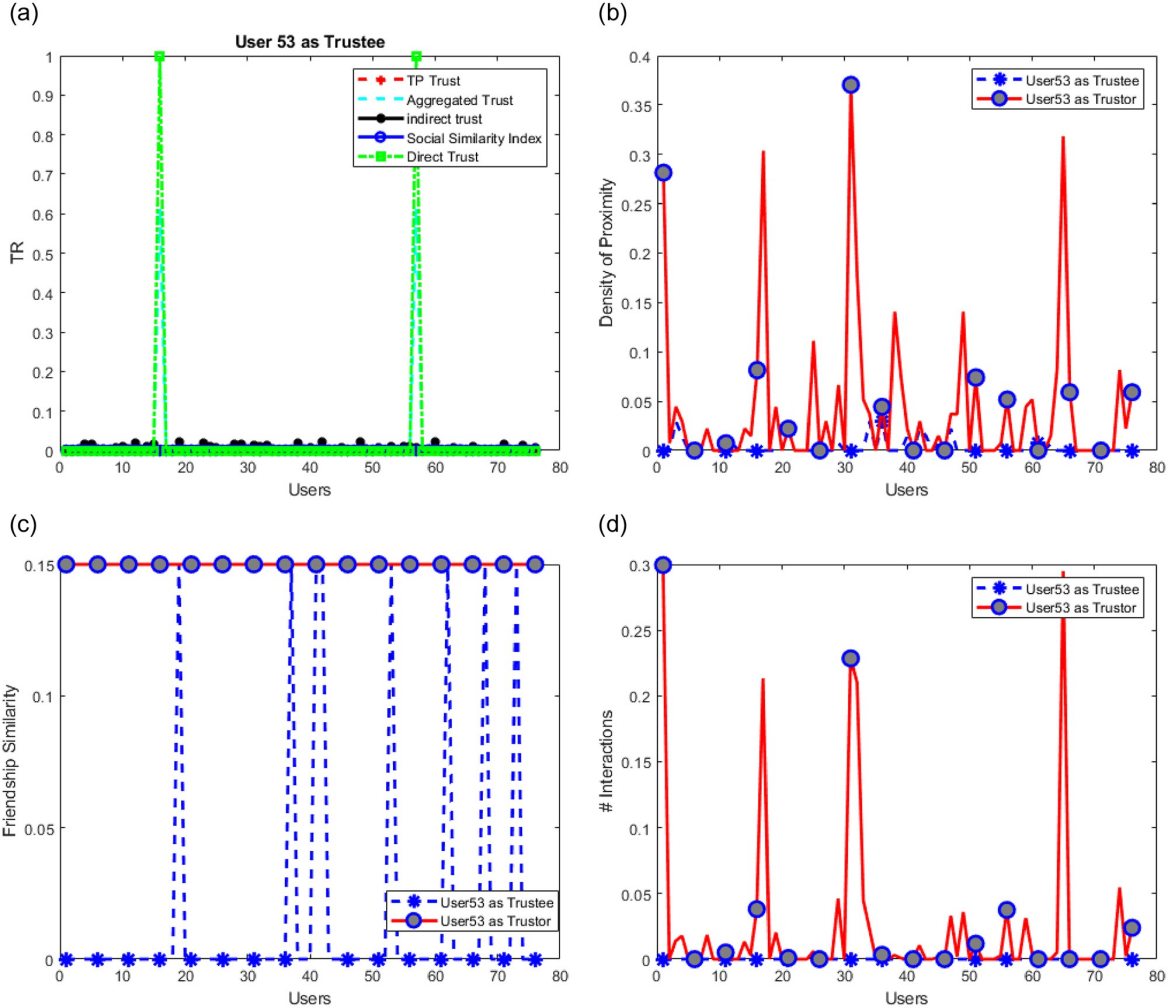
User 53 trust features before an attack. (a) user 53 predicted trust before an attack, (b) User53 proximity to other users, (c) User 53 low centrality at all to other users, (d) Low interactions with user 53 before an attack.

**Fig 15:**
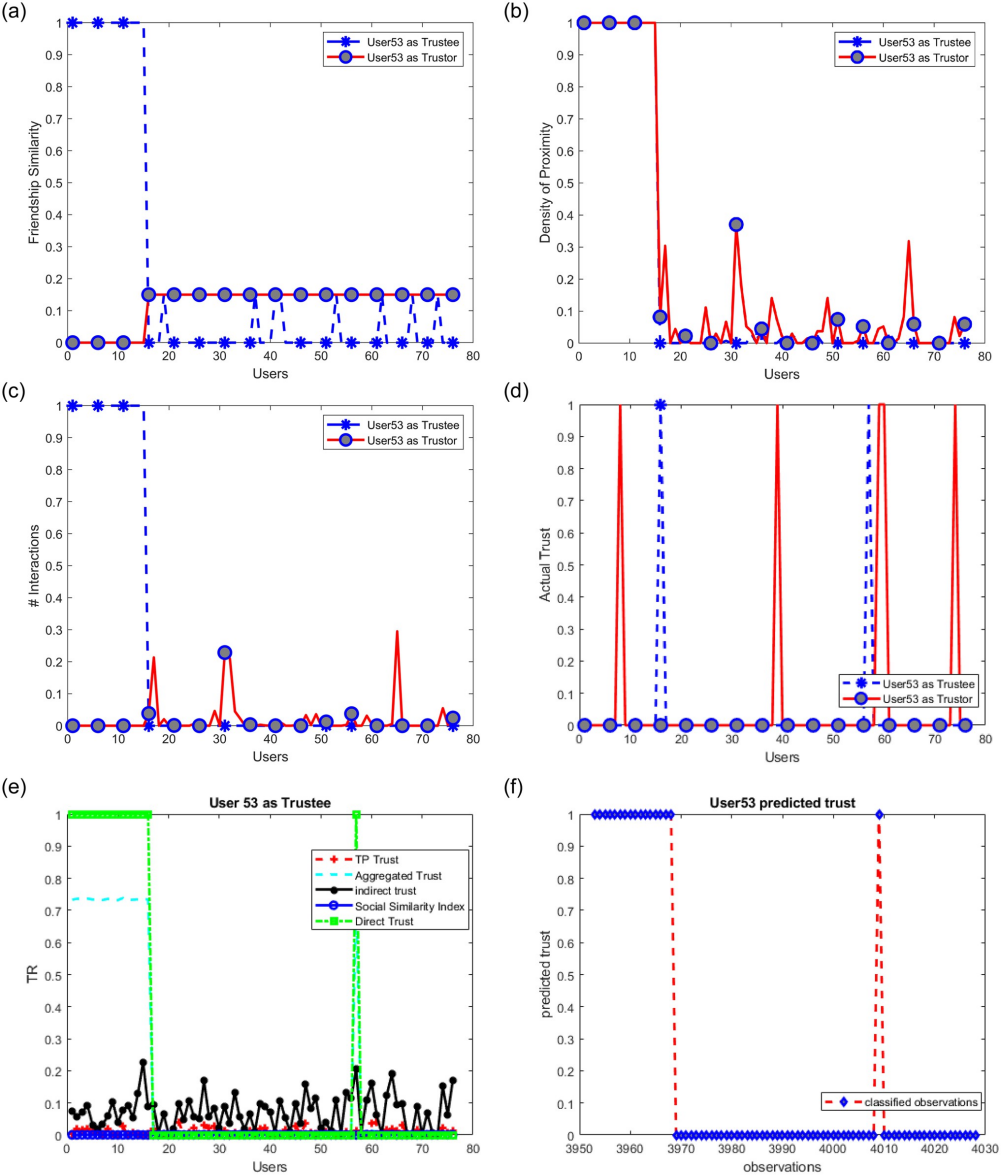
Malicious users give positive ratings to user53 (Fxy, PrZz, Prox, Cc, tr).

The use of both the highest trusted parties and social similarity is found to help reduce the effect of malicious users. In badmouthing attacks, attackers collaborate to smear, degrade, or damage the target’s reputation. In ballot stuffing, collusive users aim to deceive the trust mechanism and make it fail to accurately reflect user trustworthiness. Opportunistic attackers behave well and poorly alternately to avoid detection. In self-promotion attacks, a rogue user might boost its reputation by providing excellent ratings. Let us assume that users (1–15) be malicious users and start promoting user 53 (ballot stuffing attack) who is untrusted by all users except two (see Fig 14a). Users (1–15) are supposed therefore not to be friends with other users but with user 53 as shown in Fig 14a. At the same time, malicious users’ devices are becoming very active and appear tremendously in proximity with user 53 to support him (see Fig 14b–user 53 as trustee). However, since contacting users is not possible unless users approve, then we assume that Cc = 0. We see that despite all this fake support by those malicious users, our proposed models were not affected and user 53 remained untrusted (see Fig 15e & 15f).

Similarly, it can be shown that our model is resilient to bad-mouthing attacks which try to give users low trust values. Self-promoting attacks will not have an effect since the proposed models rely heavily on accumulating other users’ ratings and self-rating is not allowed. Opportunistic attacks, or sometimes called on/off attacks are out of the scope of this study.

### Performance on a large scale dataset

**Fig 16:**
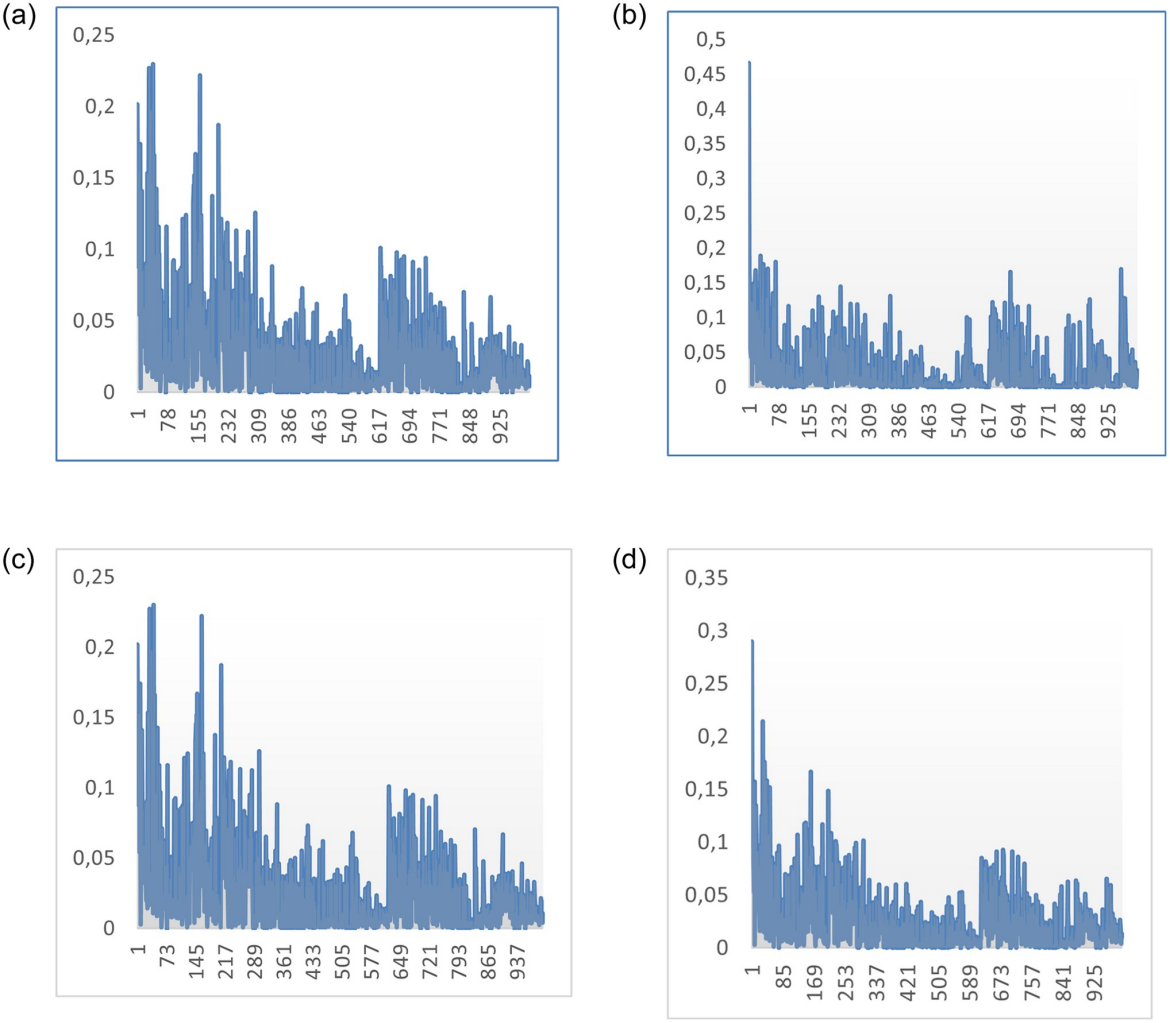
An overview of obtained trusted features for first 1000 users in the Brightkite dataset. (a) User centrality, (b) location proximity, (c) Service quality (actual ratings), (d) social similarity.

In this section, we test the performance of the proposed approaches on a large scale dataset from a well-known online location-based social network called Brightkite [55]. Brightkite enables users to communicate their whereabouts through check-ins with 58,228 nodes (users) and 214,078 edges (friendships). Only friendship, location similarities and service quality could be deduced as trust features from the dataset. Due to the highly required processing capabilities, we extracted the first 1000 users’ interactions with associated one million record observations. We used approximately 80% of the data for training purposes and 20% for testing purposes. By converting all position coordinates to kilometres and comparing each user to other users, we estimated location similarity from the check-in dataset file. If both users existed in proximity in the same month, a location similarity was noted. If this occurs multiple times within a month, it was summed up. In the end, we divided this number by the total number of location similarity occurrences a user has with all others within the same month to calculate the location similarity between Ux and Uy: LocS (Ux, Uy). Fig 16 shows an overview of obtained trusted features in the Brightkite dataset. Proposed models were implemented using this dataset, and the resulting findings are summarised in Table 2.

**Table 2:** Confusion matrix of the proposed models applied on Brightkite dataset.

	Trusted	Untrusted	Precision	Recall	Accuracy
**Proposed ML**	TP = 198562	FN = 0	1	1	1
	FP = 0	TN = 1438			
**Aggregated computed (q = 0.5)**	TP = 198562	FN = 0	1	1	1
	FP = 0	TN = 1438			
**Traditional**	TP = 198562	FN = 0	0.986	1	0.986
	FP = 1438	TN = 0			
**Direct Trust (di_tr)**	TP = 198562	FN = 0	0.99	1	0.99
	FP = 0	TN = 1438			
**Indirect Trust (In_tr)**	TP = 198562	FN = 0	0.986	1	0.99
	FP = 1438	TN = 0			
**Highly Trusted Parties (tp_tr)**	TP = 197655	FN = 907	0.987	0.996	0.98
	FP = 1358	TN = 80			

Table 2 details the proposed approaches’ performance metrics. The proposed machine learning approach and the proposed aggregated recorded 100% precision and accuracy. The three computed trust values (direct, indirect, and trust parties) showed 99% accuracy individually which are considered quite acceptable. Similar to the Crawdad dataset, the Brightkite dataset does not record dynamic user interactions at different periods and hence dynamic weighting approach was not applied.

## Conclusions

This research presented a hybrid strategy based on two models for trust computing and prediction on Internet of things scenarios utilising social similarity and machine learning techniques. We developed an asymmetrical method for computing social similarity features such as user centrality, user popularity, social contact similarities, user interactions, and user IoT device proximity. We assumed that there are so-called highly trusted parties (TP) that receive the highest scores for any of these features. We pooled three categories of computed trust: direct trust, indirect trust, and the trust of the most trusted parties. Aggregation of trust is based on a dynamic categorisation of users based on their social behaviour and previous interactions. Additionally, we demonstrated how to execute this model through the use of a cloud-based strategy. The simulation analysis indicated that the proposed machine learning and computational models performed better than related research. Additionally, it was observed that the machine learning model performed somewhat better than the computational model, which was related to the weighting variables. Additionally, it was demonstrated that, despite being less accurate in trust computation than directly computed trust, the usage of social similarity and highest trusted parties was quite effective in lowering fraudulent users’ recommendations. Additionally, a method for dynamic trust aggregation was proposed. In real-time or more dynamic datasets, the dynamic technique is shown to determine optimal aggregation weight values for each user based on his prior interactions with others which minimise the error in computation at a given time. The performance of the proposed techniques was unaffected by the size of the data, as they were evaluated on a big dataset. Additionally, it was noted that the proposed ML was unaffected by the weighting parameters chosen. In the future, we’d like to focus on opportunistic attacks while also considering more adaptive weights based on the history of user interactions. Additionally, we wish to implement the proposed approach in a real-world setting.

## Supporting information

S1 File (ZIP)
